# Supplementary material for: Impact of Pharmacist-Led Multidisciplinary Team to Attain Targeted Vancomycin Area under the Curved Monitoring in a Tertiary Care Center in Thailand
Source: Antibiotics (Basel). 2023 Feb 11;12(2):374. doi: 10.3390/antibiotics12020374 (PMC9952732; doi:10.3390/antibiotics12020374)
Supplement: Supplementary file 1 [file antibiotics-12-00374-s001.zip › antibiotics-2175923-supplementary.pdf]

Supplementary Materials

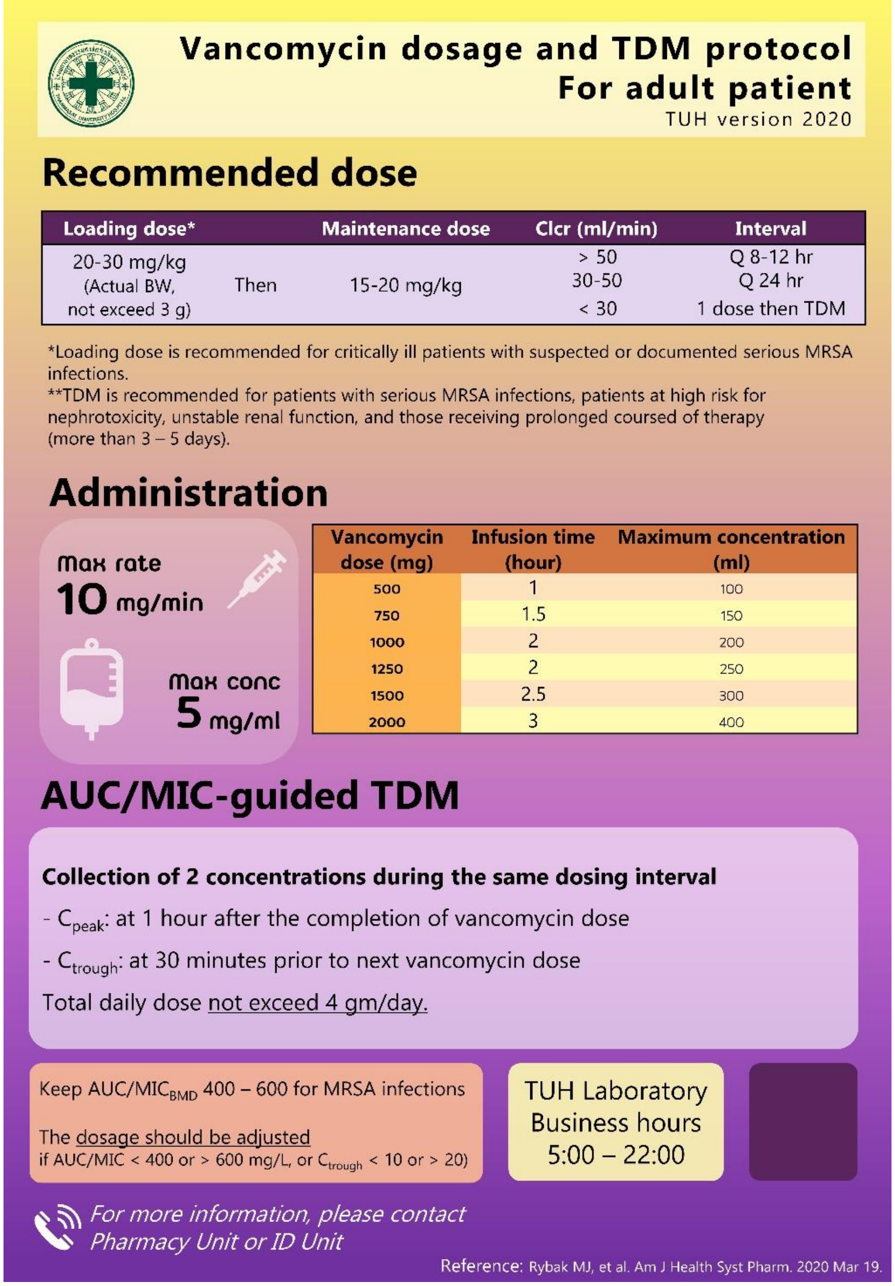

Figure S1. Thammasat university hospital vancomycin protocol.

Abbreviations: BW, body weight; CrCl, creatinine clearance; C_{peak}, peak concentration; C_{trough}, trough concentration, MRSA, methicillin-resistant *Staphylococcus aureus*; AUC, area under the curve; MIC, minimal inhibitory concentration; BMD, broth microdilution; TUH, Thammasat University Hospital

Table S2. Subgroup analysis

Outcomes	Male participant		Hypertension		Dyslipidemia		Diabetes mellitus		Bacteremia		Infections of unknown origin	
	Pre-PMT period (*n* = 56) No (%)	Post-PMT period (*n* = 51) No (%)	Pre-PMT period (*n* = 47) No (%)	Post-PMT period (*n* = 53) No (%)	Pre-PMT period (*n* = 28) No (%)	Post-PMT period (*n* = 36) No (%)	Pre-PMT period (*n* = 28) No (%)	Post-PMT period (*n* = 26) No (%)	Pre-PMT period (*n* = 21) No (%)	Post-PMT period (*n* = 13) No (%)	Pre-PMT period (*n* = 36) No (%)	Post-PMT period (*n* = 31) No (%)
Achieved therapeutic target	26 (46.43)	33 (64.71)<br>(*p*=0.058)	16 (34.04)	34 (64.15)<br>(*p*=0.003)	11 (39.29)	25 (69.44)<br>(*p*=0.016)	7 (26.92)	15 (53.57)<br>(*p*=0.046)	6 (46.15)	15 (71.143)<br>(*p*=0.168)	13 (36.11)	20 (64.52)<br>(*p*=0.020)
Calculated AUC, mean mg·hr/L ± SD	595.96 ± 170.12	577.61 ± 184.20<br>(*p*=0.593)	651.29 ± 177.50	606.39 ± 189.38<br>(*p*=0.226)	665.53 ± 202.76	556.33 ± 167.63<br>(*p*=0.022)	629.57 ± 188.77	619 ± 215<br>(*p*=0.857)	555.45 ± 225.22	500.16 ± 136.03<br>(*p*=0.377)	638.43 ± 182.54	582.84 ± 176.71<br>(*p*=0.212)
Calculated AUC range												
AUC less than 400 mg×hr/L	4(7.14)	4 (7.84)<br>(*p*=1.000)	4 (8.51)	1 (1.89)<br>(*p*=0.184)	2 (7.14)	3 (8.33)<br>(*p*=0.860)	3 (11.54)	2 (7.14)<br>(*p*=0.663)	2 (15.38)	4 (19.05)<br>(*p*=1.000)	3 (8.33)	1 (3.23)<br>(*p*=0.618)
AUC within range (400-600 mg×hr/L)	26 (46.43)	33 (64.71)<br>(*p*=0.058)	16 (34.04)	34 (64.15)<br>(*p*=0.003)	11 (39.29)	25 (69.44)<br>(*p*=0.016)	7 (26.92)	15 (53.57)<br>(*p*=0.046)	6 (46.15)	15 (71.43)<br>(*p*=0.168)	13 (36.11)	20 (64.52)<br>(*p*=0.020)
AUC more than 600 mg×hr/L	26 (46.43)	14 (27.45)<br>(*p*=0.043)	27 (57.45)	18 (33.96)<br>(*p*=0.018)	15 (53.57)	8 (22.22)<br>(*p*=0.010)	16 (61.54)	11 (39.29)<br>(*p*=0.102)	5 (38.46)	2 (9.52)<br>(*p*=0.079)	20 (55.56)	10 (32.26)<br>(*p*=0.056)
Trough concentration, mean mg/L ± SD	15.38 ± 6.67	13.96 ± 4.26<br>(*p*=0.198)	18.08 ± 6.46	15.38 ± 6.69<br>(*p*=0.043)	17.25 ± 5.25	15.21 ± 7.29<br>(*p*=0.217)	16.65 ± 5.32	15.60 ± 4.31<br>(*p*=0.429)	16.11 ± 8.72	16.05 ± 9.07<br>(*p*=0.985)	15.58 ± 6.18	13.88 ± 5.02<br>(*p*=0.227)
Clinical cure	37 (66.07)	48 (94.12)<br>(*p*<0.001)	33 (70.21)	50 (94.34)<br>(*p*=0.002)	19 (67.86)	33 (91.67)<br>(*p*=0.023)	16 (61.54)	26 (92.86)<br>(*p*=0.008)	4 (30.77)	18 (85.71)<br>(*p*=0.002)	27 (75)	30 (96.77)<br>(*p*=0.016)
30-days infectious diseases mortality	10 (17.86)	3 (5.88)<br>(*p*=0.077)	8 (17.02)	0 (0)<br>(*p*=0.002)	4 (14.29)	2 (5.56)<br>(*p*=0.391)	5 (19.23)	1 (3.57)<br>(*p*=0.095)	4 (30.77)	1 (4.76)<br>(*p*=0.059)	4 (11.11)	1 (3.23)<br>(*p*=0.363)
Nephrotoxicity event	2 (3.57)	3 (5.88)<br>(*p*=0.668)	1 (2.13)	3 (5.66)<br>(*p*=0.620)	0 (0)	2 (5.56)<br>(*p*=0.500)	0 (0)	1 (3.57)<br>(*p*=0.298)	0 (0)	2 (9.52)<br>(*p*=0.513)	1 (2.78)	1 (3.23)<br>(*p*=1.000)
Proportion of patient who achieved therapeutic range within 48 hours	13 (23.21)	16 (31.37)<br>(*p*=0.343)	10 (21.28)	21 (39.62)<br>(*p*=0.048)	6 (21.43)	16 (44.44)<br>(*p*=0.054)	3 (11.54)	7 (25)<br>(*p*=0.298)	5 (38.46)	6 (28.57)<br>(*p*=0.709)	4 (11.11)	11 (35.48)<br>(*p*=0.021)
Time to target, median days (IQR)	3 (2-4)	3 (1-5)<br>(*p*=0.968)	3 (2-6)	2 (1-4)<br>(*p*=0.467)	3 (1-5)	2 (1-4)<br>(*p*=0.527)	4 (2-9)	3 (2-6)<br>(*p*=0.374)	2 (1-2)	3 (1-5)<br>(*p*=0.175)	3 (3-4)	3 (2-4)<br>(*p*=0.466)
Vancomycin consumption, median DDD per 1000 patient-day (IQR)	7.75 (5.25-12.75)	10 (5.5-14)<br>(*p*=0.255)	7.88 (5-12.88)	7 (4.75-10.25)<br>(*p*=0.030)	6.88 (5.13-10)	10.25 (5.25-14.75)<br>(*p*=0.123)	5.75 (4.25-9.25)	7.5 (5.13-13)<br>(*p*=0.197)	4.25 (2.75-5.5)	7 (5-10.25)<br>(*p*=0.013)	8.13 (4.75-18)	11 (8-17)<br>(*p*=0.180)
Length of stay, median days (IQR)	29 (21.5-54.5)	33 (20-55)<br>(*p*=0.803)	30 (16-43)	33 (19-48)<br>(*p*=0.229)	31.5 (22-43.5)	32 (18.5-46)<br>(*p*=0.919)	29.5 (12-43)	32 (18.5-49)<br>(*p*=0.436)	22 (20-45)	34 (19-48)<br>(*p*=0.684)	27.5 (14.5-42)	27 (19-42)<br>(*p*=0.619)

AUC, Area Under the Curve; DDD, defined daily dose; IQR, interquartile range; SD, standard deviation
